# Wearable Hyperspectral Photoplethysmography Allows Continuous Monitoring of Exercise‐Induced Hypertension

**DOI:** 10.1002/advs.202417625

**Published:** 2025-04-25

**Authors:** Jung‐Woo Park, Jaehyeok Park, Jaehun Jeon, Seongok Chae, Gi Beom Kim, Geonhui Han, Hyung‐Soon Park, Yong Jeong, Ki‐Hun Jeong

**Affiliations:** ^1^ Department of Bio and Brain Engineering Korea Advanced Institute of Science and Technology (KAIST) 291 Daehak‐ro, Yuseong‐gu Daejeon 34141 South Korea; ^2^ KAIST Institute for Health Science and Technology (KIHST) Korea Advanced Institute of Science and Technology (KAIST) 291 Daehak‐ro, Yuseong‐gu Daejeon 34141 South Korea; ^3^ Department of Mechanical Engineering Korea Advanced Institute of Science and Technology (KAIST) 291 Daehak‐ro, Yuseong‐gu Daejeon 34141 South Korea; ^4^ Program of Brain and Cognitive Engineering Korea Advanced Institute of Science and Technology (KAIST) 291 Daehak‐ro, Yuseong‐gu Daejeon 34141 South Korea

**Keywords:** continuous blood pressure monitoring, hyperspectral, microspectrometer, photoplethysmography, solid immersion grating

## Abstract

Continuous blood pressure (BP) monitoring is essential for cardiovascular health, yet current BP sensors face cuff‐dependent limitations. Cuff‐free alternatives still suffer from discomfort and discontinuous measurement. Here a wearable hyperspectral photoplethysmography (HS‐PPG) is reported for continuous and nonconscious BP monitoring. The HS‐PPG module integrates an ultrathin and high‐resolution double‐folded solid immersion grating microspectrometer (DFSIG‐µSPEC) with a white light LED. DFSIG‐µSPEC shows an average spectral resolution of 3.4 nm for 550–800 nm in the operational range. The HS‐PPG module has a compact physical dimension of 8 mm × 16 mm × 24 mm, suitable for wrist‐wearable configuration. The PPG waveforms contain 50 spectral bands, achieving precise measurement of arteriolar pulse transit time (aPTT). The diastolic and systolic BPs are precisely estimated with R‐values of 0.92 and 0.96, and mean absolute differences (MAD) of 1.20 and 0.40 mmHg with the 2‐element Windkessel model, respectively. Further, the BP is continuously measured with heart rate (HR) and respiratory exchange ratio (RER) with exercise‐induced hypertension. Continuous monitoring of systolic blood pressure (SBP) exhibits immediate responses during hemodynamic changes, with the physiological parameters of SBP, HR, and RER during exercise and recovery. The wearable HS‐PPG clearly supports the strong potential for high‐fidelity continuous BP monitoring.

## Graphical abstract

A wearable hyperspectral photoplethysmography (HS‐PPG) is reported for continuous blood pressure (BP) monitoring. The HS‐PPG module integrates an ultrathin double‐folded solid immersion grating microspectrometer (DFSIG‐µSPEC), achieving a high spectral resolution of 3.4 nm. The HS‐PPG allows precise PPG waveform measurement with hyperspectral range, estimating BP with high accuracy (R^2^ up to 0.96). The HS‐PPG with demonstrates strong potential for cuff‐free and continuous BP monitoring.

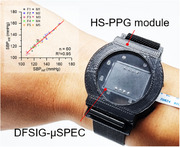

## 1 Introduction

Continuous blood pressure (BP) monitoring provides accurate insights into BP dynamics, playing a crucial role in personalized healthcare.^[^ ^1^, ^2^, ^3^ ^]^ Early detection of hypertension allows precise assessments of therapeutic efficacy,^[^ ^4^, ^5^ ^]^ and the medication compliance enhances patient's treatments.^[^ ^6^, ^7^ ^]^ In addition, continuous BP monitoring supports remote patient management, offering telehealth vital for those with mobility restrictions.^[^ ^8^, ^9^, ^10^ ^]^ Tracking BP levels proactively addresses cardiovascular conditions, allowing personalized healthcare based on comprehensive and individualized data.^[^ ^11^, ^12^ ^]^ Conventional methods such as oscillometry^[^ ^13^, ^14^ ^]^ and auscultation^[^ ^15^, ^16^ ^]^ rely on the air cuff but show some technical limitations due to restricted contact and discontinuous measurements.^[^ ^17^, ^18^ ^]^ Ultrasound,^[^ ^19^, ^20^ ^]^ strain sensors,^[^ ^21^, ^22^ ^]^ or tonometry^[^ ^23^ ^]^ provide cuff‐free options, yet achieving fully unobtrusive and continuous BP measurement still remains a challenging task.^[^ ^24^ ^]^

Arterial pulsatile waveforms recently emerge as a non‐invasive method for BP estimation.^[^ ^25^, ^26^ ^]^ Conventional pulse transit time (PTT), relying on the time difference between electrocardiogram (ECG) and photoplethysmography (PPG) waveforms, reduces physical restrictions, yet ECG electrode attachment still constrains the comfort of continuous monitoring.^[^ ^27^, ^28^, ^29^, ^30^ ^]^ Arteriolar PTT (aPTT) unlike PTT analyzes multi‐wavelength PPG waveforms from vascular layers at a single location, allowing entirely unobtrusive BP measurement.^[^ ^31^, ^32^ ^]^ However, the absence of gold standard signals such as ECG waveforms in PTT compromises the estimation accuracy due to the temporal division of multi‐wavelength PPG waveforms.^[^ ^33^, ^34^ ^]^ As a result, alternative approaches, particularly within the spectrum of PPG waveforms without reliance on external standard signal, are crucial for enhancing the accuracy and reliability of continuous BP monitoring.

## 2 Results

**Figure 1:**
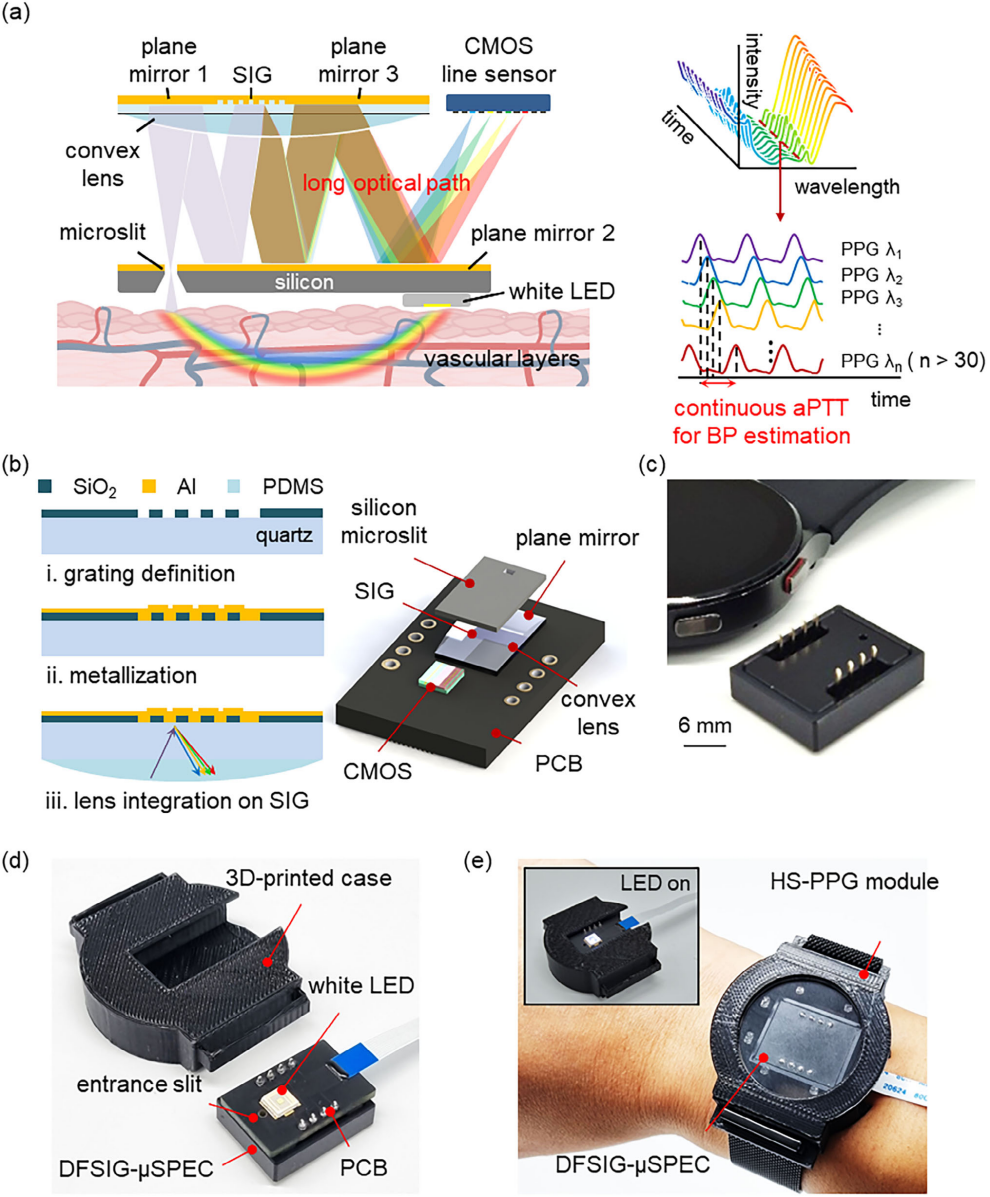
A schematic illustration for continuous monitoring of blood pressure using wearable hyperspectral photoplethysmography (PPG) sensor with double‐folded solid immersion grating microspectrometer (DFSIG‐µSPEC). a) The wearable hyperspectral PPG module comprises DFSIG‐µSPEC and a white LED. Light emitted from the LED penetrates the skin and undergoes reflection. The reflected light passes through a silicon microslit, dispersed through the solid immersion grating (SIG), undergoing multiple reflections and refractions with plane mirrors and a convex lens. Dispersed light delivers PPG signals on a CMOS line sensor at the level of hyperspectral range. Measured spectra are accumulated into 3D PPG datasets over time. Hyperspectral PPG signals allow the precise calculation of continuous arteriolar pulse transit time (aPTT) for blood pressure estimation. b) The microfabrication steps and module integration of DFSIG‐µSPEC. The SIG is microfabricated by using wafer stepper lithography and reactive ion etching (step i), and metallized with an aluminum thin layer (step ii). A convex lens is molded with PDMS (step iii). The SIG and a CMOS line sensor are mounted on a printed circuit board (PCB), and integrated with a silicon microslit. c) An optical image of DFSIG‐µSPEC, fully‐packaged with a module thickness of 5.8 mm. d,e) Captured optical image for wrist‐wearable configuration of (d) fully‐integrated HS‐PPG module and (e) white LED module.

Here we report wearable hyperspectral photoplethysmography (HS‐PPG) for continuous BP monitoring with unrestricted contact and hands‐free operation. The HS‐PPG module comprises an ultrathin double‐folded solid immersion grating microspectrometer (DFSIG‐µSPEC) and a white light LED (**Figure** **1**a; Figure S1, Supporting Information). DFSIG‐µSPEC disperses light with high angular dispersion, based on the solid immersion grating (SIG) structure.^[^ ^35^ ^]^ DFSIG‐µSPEC continuously measures PPG waveforms at different wavelengths, which are reflected from the vascular layers under white light LED illumination. Reflected light is entered through a silicon microslit, and dispersed through the SIG, and captured by the CMOS line sensor. Multiple reflections between two plane mirrors increase the optical path and reduce the overall thickness of the microspectrometer. Measured HS‐PPG waveforms are accumulated into a time‐lapsed spectral dataset, allowing the calculation of a finely aligned continuous aPTT value.

The SIG of DFSIG‐µSPEC was microfabricated on a wafer scale (Figure 1b, **left**). The grating structures with a series of parallel lines were first defined by using plasma enhanced chemical vapor deposition (PECVD), wafer stepper lithography, and reactive ion etch (RIE) (step i). A 100 nm‐thick aluminum layer was then thermally evaporated on top of the grating structures (step ii). The convex lens was molded and integrated on the bottom (step iii). The SIG has a binary‐phase structure with a period of 1.25 µm, a height of 100 nm, and a duty cycle of 0.4 (Figure S2, Supporting Information). A silicon microslit was also microfabricated by using double‐side deep reactive ion etching (DRIE) with a rectangular aperture of 15 µm × 200 µm (Figure S1, Supporting Information). The SIG and the silicon microslit were precisely aligned with identical physical dimensions (Figure 1b, **right**). The silicon microslit was mounted onto the printed circuit board (PCB) with the CMOS line sensor (S13131‐512, Hamamatsu Photonics) and packaged in an anodized aluminum case. A fully‐packaged DFSIG‐µSPEC has a compact physical dimension of 5.8 mm × 16 mm × 24 mm (Figure 1c). DFSIG‐µSPEC was further utilized for HS‐PPG waveform measurement and continuous BP estimation. HS‐PPG module was integrated for the PPG waveform measurement at the wrist (Figure 1d,e). DFSIG‐µSPEC and white LED were mounted on a PCB, and connected to the data acquisition (DAQ) board with flexible cable, and housed in a watch‐type 3D‐printed case. The source‐detector distance, i.e., the distance between the center of the white LED and the entrance slit of DFSIG‐µSPEC, was set to 3 mm to cover the depth to the arteriolar and artery layers (Figure S3, Supporting Information, see Experimental Section).

**Figure 2:**
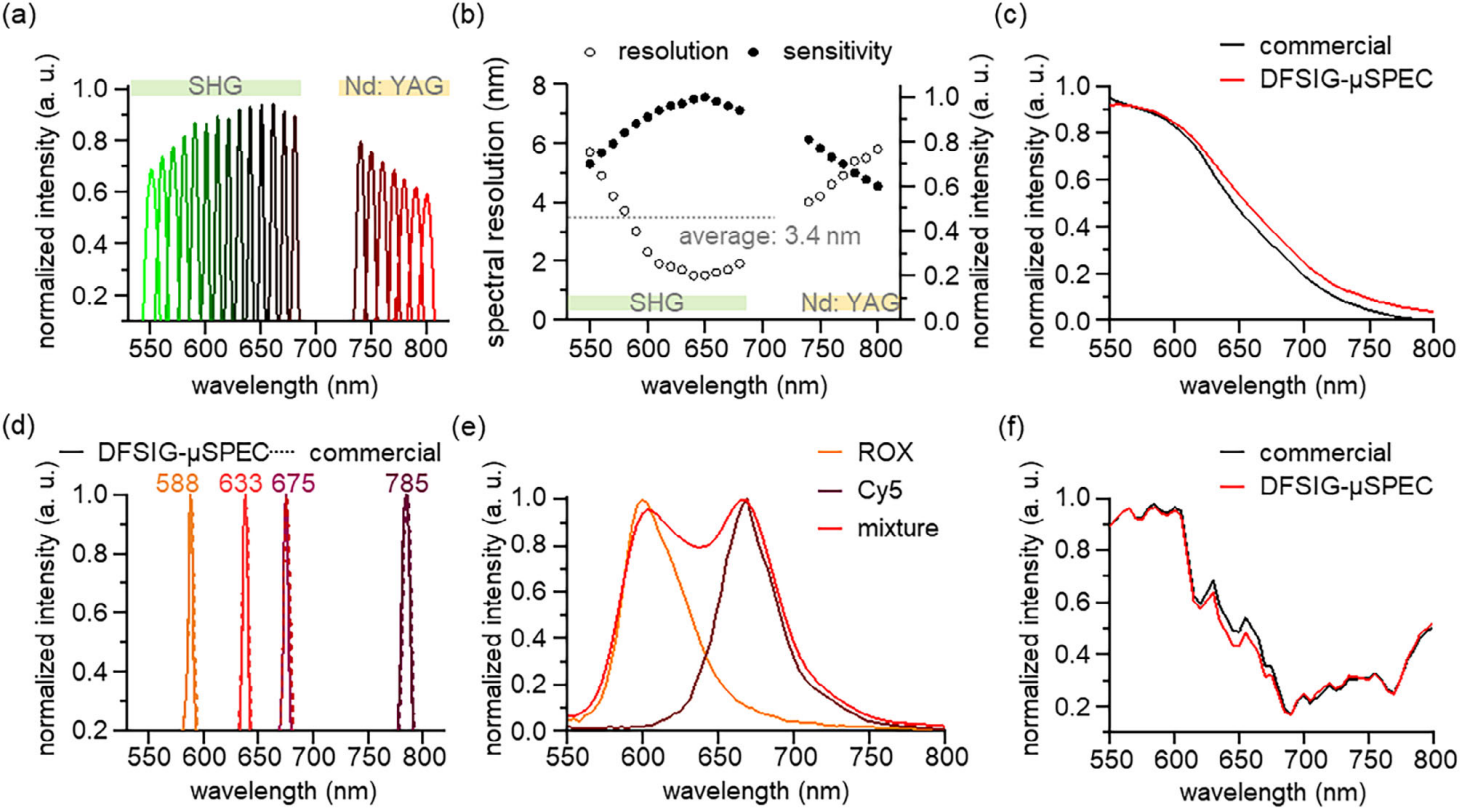
Spectral resolution of DFSIG‐µSPEC. a) Output spectra of Nd: YAG tunable laser measured by DFSIG‐µSPEC. Each spectrum exhibits a sharp spectral peak in the operational range of DFSIG‐µSPEC. b) Spectral resolution and optical sensitivity. DFSIG‐µSPEC exhibits an average resolution of 3.4 nm and a relative sensitivity of over 60% of the maximum. c) Spectrum measurement of white LED light, where both the commercial spectrometer and DFSIG‐µSPEC are nearly identical. d) Output spectra of narrow band LEDs with spectral peaks at 588, 633, 675, and 785 nm. Measured spectra are well‐matched with those from the commercial spectrometer. e) Emission spectra of 1 *µM*‐concentration of ROX, Cy5, and mixed solutions. Two spectral peaks exhibit well‐defined peaks with a 78% overlap intensity. f) Reflectance spectra at the wrist skin. The measured spectrum of DFSIG‐µSPEC exhibits similar spectral profiles of the reference spectrum.

The spectral resolution of DFSIG‐µSPEC was evaluated within the operational range (**Figure** **2**). The spectra were measured by using Nd: YAG tunable laser (NT341A, EKSPLA) with 10 nm wavelength interval (Figure S4, Supporting Information). Laser light at different wavelengths was passed through ND filter, aperture (AP), fiber‐coupled collimator (FCC), and objective lens (OBJ) with a numerical aperture (NA) of 0.2. Focused light was then injected into DFSIG‐µSPEC and visualized into spectral data with DAQ software (LabVIEW DAQmx module, National Instrument). The output spectra exhibit sharp Lorentzian distributions at the operational range between 550 and 800 nm (Figure 2a). Note that DFSIG‐µSPEC has a wavelength gap between Nd: YAG radiation (over 740 nm) and second harmonic generation (SHG) (550–680 nm). The spectral resolution and optical sensitivity were calculated from the output spectra (Figure 2b). Quantitative analysis reveals an average spectral resolution of 3.4 nm and a relative sensitivity of over 60% of the maximum. In addition, DFSIG‐µSPEC exhibits remarkable fidelity in continuous measurement of LED spectrum. Notably, DFSIG‐µSPEC demonstrates the continuous white LED emission spectrum and the output spectra of 588, 633, 675, and 785 nm LEDs with a narrow band, with nearly identical compared to the conventional spectrometer (Figure 2c,d). The emission spectra of 1 *µM* 6‐Carboxyl‐Rhodamine (ROX), Cyanine 5 (Cy5), and both mixed solutions of fluorescent dyes were also measured with DFSIG‐µSPEC (Figure 2e). DFSIG‐µSPEC clearly distinguishes two emission spectra of fluorescent dyes, with a 78% overlap intensity between two adjacent peaks at 604 and 670 nm. Furthermore, DFSIG‐µSPEC measured the diffuse reflectance spectrum of the wrist skin (Figure 2f). Reflection spectra exhibits similar spectral profiles at the operational range, underscoring the high‐resolution of the microspectrometer for the BP estimation performance.

**Figure 3:**
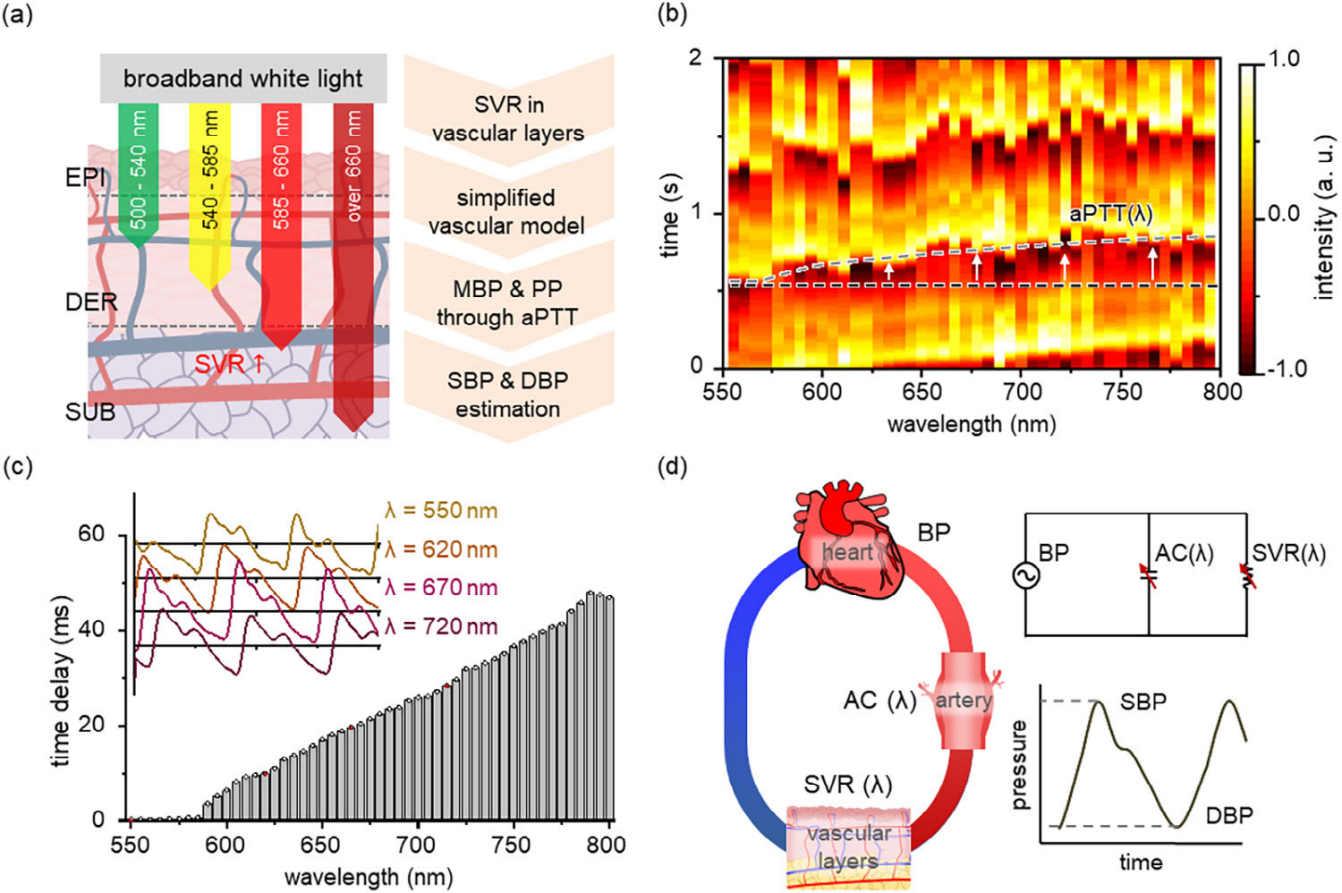
Hyperspectral PPG (HS‐PPG) waveform measurement for continuous blood pressure estimation. a) Blood pressure estimation flowchart. Light is passed through the vascular layers of the epidermis (EPI), dermis (DER), and subcutaneous tissue (SUB) and absorbed in the blood vessels. Systemic vascular resistance (SVR), i.e., a measure of resistance to blood flow, differs from the vascular layers. A vascular model is simplified with different SVR values. Systolic/diastolic blood pressures are finally estimated from the SVR values after calculation of the mean blood pressure (MBP) and pulse pressure (PP). b) Time‐lapsed HS‐PPG waveforms (sampling rate: 1 *kHz*). Continuous aPTT values are calculated using the time difference for the shortest wavelength of 550 nm. c) The time delay between the PPG peaks using the 550 nm peak as the reference and some examples of PPG waveforms. The PPG waveform exhibits higher time delay as the wavelength increases, due to deeper penetration depth. d) A simplified vascular model for continuous aPTT. The vascular layers are replaced by an equivalent electrical circuit with SVR and arterial compliance (AC), which are affected by the wavelength. The simplified model estimates the systolic and diastolic blood pressure levels.

A comprehensive BP estimation procedure is based on the simplified vascular layers (**Figure** **3**a). Light is passed through the vascular layers of the epidermis, dermis, and subcutaneous tissue, and absorbed by the blood vessels. The systemic vascular resistance (SVR), an indicator of resistance to blood flow, varies across the vascular layers. A simplified vascular model with different SVR values is used to estimate systolic and diastolic BPs from the calculated mean blood pressure (MBP) and pulse pressure (PP). The measured spectra are transmitted through the DAQ board and digitized with a sampling acquisition rate of 500 *kHz*. Each PPG waveform has a sampling rate of 1 *kHz* with a measurement time of 10 s (Figure S5, Supporting Information). Continuous aPTT is calculated from the time differences between PPG peaks, using the 550 nm peak as the reference (Figure 3b). The average time delays relative to the 550 nm PPG peak are exhibited after applying digital filtration and peak extraction from 50 PPG waveforms (Figure 3c). Time delay steadily increases as the wavelength of the PPG waveforms increases, due to the deeper penetration of light to the vascular layers. Continuous aPTT within the operational range is then used to acquire blood pressure levels. A simplified vascular model with continuous aPTT is represented by an equivalent electrical circuit where SVR and arterial compliance (AC) depend on wavelength (Figure 3d). A vascular model allows aPTT to indicate BP variations based on the two‐element Windkessel model.^[^ ^36^ ^]^ In the BP estimation based on the Windkessel model, MBP and PP are estimated from the scatter plot of HR(λ) and aPTT(λ). Finally, SBP and diastolic blood pressure (DBP) are calculated from these values (See Experimental Section).

**Figure 4:**
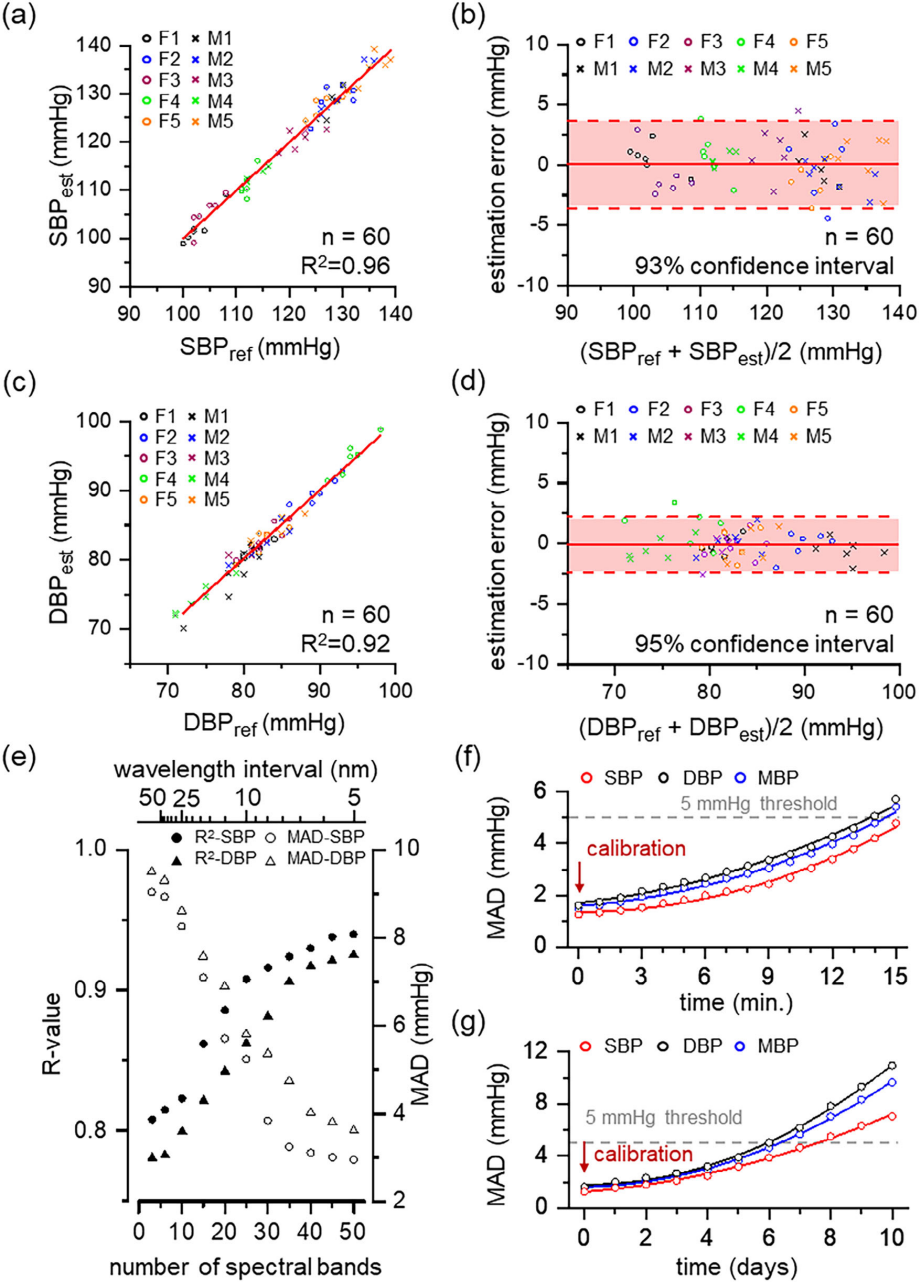
Comparative analysis of blood pressure measurement in normotensive states. a) A strong linear correlation of DBP values from cuff‐based BP sensor and HS‐PPG module. The estimated BP is monitored through HS‐PPG module, whereas the reference BP is measured by a cuff‐based BP sensor. DBPs show high linearity with an R‐value of 0.92 and the mean absolute difference (MAD) of 1.20 mmHg. (n = 60, *p* < 0.05) b) The Bland–Altman plot for DBP values. c) A strong linear correlation of SBP values from cuff‐based BP sensor and HS‐PPG module. SBPs also show high linearity with an R‐value of 0.96 and the MAD of 0.40 mmHg. (n = 60, *p* < 0.05) d) The Bland–Altman plot for SBP values. The mean estimation error is ‐0.07 mmHg for DBP and 0.04 mmHg for SBP, with confidence intervals over 93% for each. e) The R‐value and MAD in HS‐PPG waveform depending on the number of spectral bands (the operational range over the wavelength interval), limited by the spectral resolution of DFSIG‐µSPEC. High spectral resolution significantly enhances estimation reliability, increasing the R‐value of SBP from 0.81 to 0.94 and DBP from 0.78 to 0.92. MAD also reduces from 9.04 to 2.94 mmHg of SBP and 9.50–3.62 mmHg of DBP. f) Short‐term signal stability at frequent intervals. MADs show a slight increase in DBP and SBP values by 3.53 and 4.1 mmHg after 15 min, respectively. g) Long‐term signal drift. MAD slightly increases by 5.79 mmHg in SBP and 9.31 mmHg in DBP after 10 days. Both short‐term and long‐term stability ensure precise BP estimation with the HS‐PPG module without frequent calibration.

The performance of BP estimation was evaluated in normotensive subjects using a cuff‐based BP sensor and HS‐PPG module (**Figure** **4**; Figure S7, Supporting Information). The DBP values show a strong linear correlation of continuous BP estimation with the estimated DBP from the HS‐PPG module closely matching the reference DBP from the sensor (Figure 4a). Two DBPs exhibit high reliability with an R‐value of 0.92 and a mean absolute difference (MAD) of 1.20 mmHg. The Bland–Altman plot for both DBP values validates the estimation accuracy with a mean estimation errors of −0.07 mmHg and the confidence intervals of 95% (Figure 4b). Similarly, SBP values have an R‐value of 0.96 and a MAD of 0.40 mmHg (Figure 4c), with the Bland–Altman plot showing a mean error of 0.04 mmHg and 93% confidence intervals (Figure 4d). Note that 60 data points from the HS‐PPG module were collected over 10 min, with 2‐min intervals, to align with the temporal resolution of the cuff‐based BP sensor for comparison. Such precise estimation of SBP and DBP results from the high spectral resolution of DFSIG‐µSPEC. In other words, both the R‐value and MAD for SBP and DBP substantially improve as the number of PPG waveforms (Figure 4e). The hyperspectral range of PPG waveforms improves estimation reliability by comparing HS‐PPG with the BP estimation using wavelength of PPG waveforms of 530, 660, and 785 nm, which are commonly used in multi‐wavelength PPG. R‐values increase from 0.81 to 0.94 and DBP from 0.78 to 0.92 as the number of spectral bands (the operational range over the wavelength interval) increases. MADs also reduce from 9.04 to 2.94 mmHg for SBP and 9.50–3.62 mmHg for DBP. These improvements are largely attributed to the precise measurement of the hyperspectral range in PPG waveforms. HS‐PPG based BP estimation exhibits improved quantitative accuracy compared to the multi‐PPG method due to the simultaneous measurement of hyperspectral range of PPG waveforms with DFSIG‐µSPEC. Moreover, the short‐term signal stability is significantly improved of HS‐PPG (Figure 4f). After initial calibration, MADs show slight increases in both DBP and SBP values after 15 min, from 1.62 to 5.72 mmHg and 1.26–5.72 mmHg, respectively. Long‐term signal drift shows slightly different aspect with repeatability demonstration (Figure 4g). MADs at SBP and DBP slightly increased by 5.79 and 9.31 mmHg after 10 days, satisfying the standard level of the BP estimation (from *IEEE 1708 standard: Grade A*). Note that MADs for both SBP and DBP are low enough than 5 mmHg for the home BP standard level.^[^ ^37^ ^]^ The HS‐PPG module provides precise BP estimation with minimal need for re‐calibration, unlike conventional BP estimations that require frequent calibration to maintain accuracy.^[^ ^38^ ^]^

**Figure 5:**
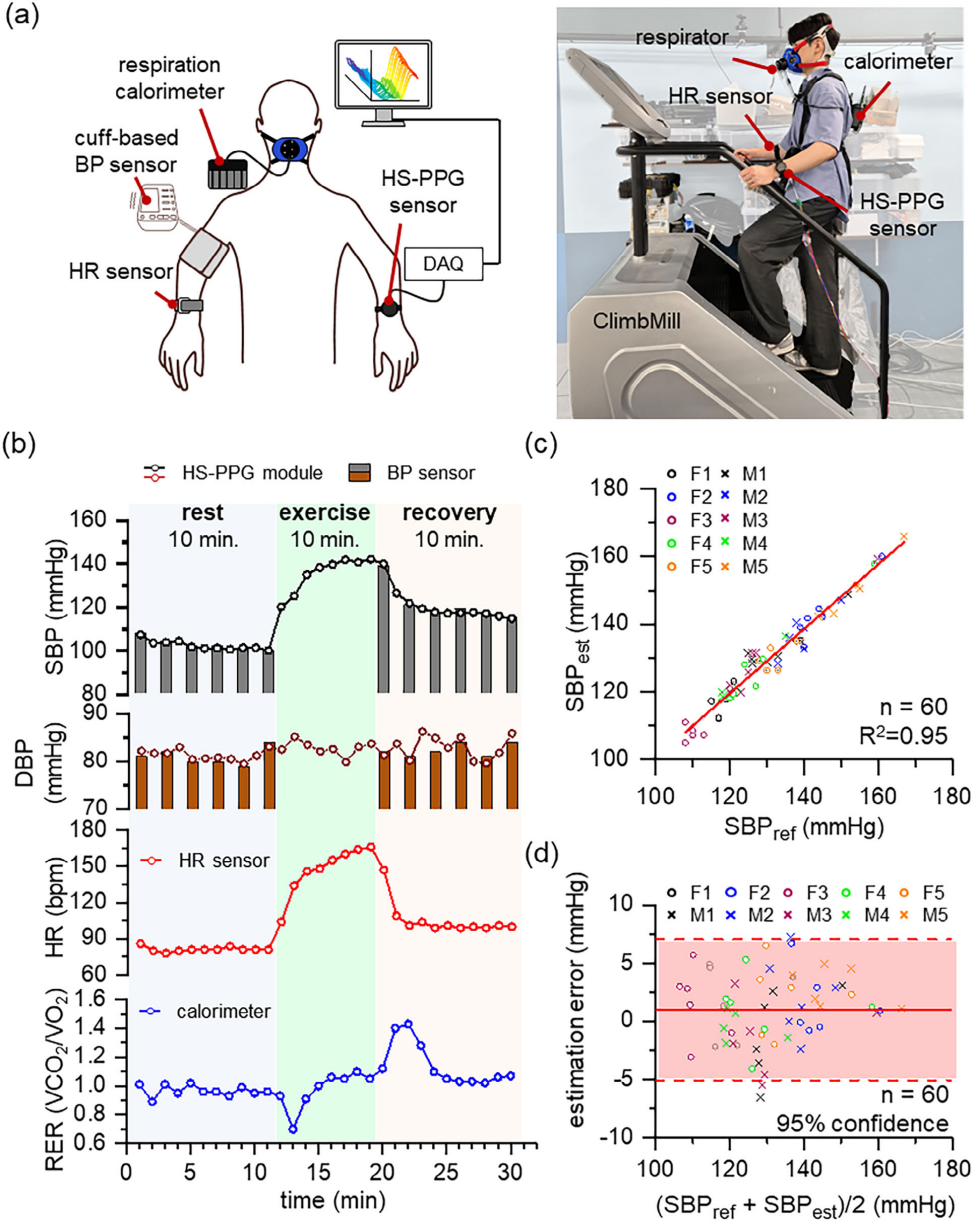
Continuous BP monitoring during hemodynamic changes. a) Experimental setup for continuous BP monitoring. HS‐PPG waveforms, BP, heart rate (HR), and respiratory exchange ratio (RER) were simultaneously monitored with respective sensors. b) A representative example of SBP, HR, and RER signals, recorded from a single subject. All the experiments are performed with three steps (10‐min rest, 10‐min exercise, and 10‐min recovery). Both reference and estimated SBPs significantly increase during exercise and decreased during recovery. SBP is measured at 1‐min interval during exercise with the HS‐PPG module, unlike cuff‐based BP sensor. Note that cuff‐based BP is inaccurate during exercise due to motion artifact. HR sharply increases during exercise and decreases during recovery, similar to SBP. RER slightly decreases immediately after exercise and then overshoots during recovery. HS‐PPG module clearly exhibits immediate responses of SBP during hemodynamic changes like the performance of the HR sensor or calorimeter. c) A strong linear correlation between reference and estimated SBPs during the recovery step. Estimated SBP exhibits a high correlation (R^2^ = 0.95) with the reference SBP (n = 60, *p* < 0.05). d) The Bland–Altman plot for reference and estimated SBPs. The mean estimation error is 0.98 mmHg with confidence intervals of 95%.

Continuous BP monitoring was further evaluated using the wearable HS‐PPG module during exercise‐induced hypertension (EIH) (**Figure** **5**a). Each of the ten normotensive subjects free of cardiovascular diseases was equipped with a cuff‐based BP sensor, HS‐PPG module, heart rate (HR) sensor, and respiration calorimeter. The clinical experiment was conducted in three 10‐min stages: rest (pre‐exercise), climbing exercise, and recovery (post‐exercise) (See Experimental Section). SBPs, HR, and respiratory exchange ratio (RER) were concurrently monitored across all the sequential stages (Figure 5b; Figures S8–S17, Supporting Information). The SBP values significantly rise during exercise and then decrease during recovery. The HS‐PPG module continuously tracks dynamic changes in SBP, closely aligning with BP sensor measurements, and also monitors SBP during exercise, where the BP sensor is impeded by motion artifacts. The heart rate varies with SBP in a similar fashion, demonstrating a remarkable physiological coherence during physical activities. Moreover, RER, tracked by the respiration calorimeter, exhibits a sudden drop immediately after exercise followed by an overshoot during the recovery phase. This RER pattern reflects the metabolic equilibrium of the human body and is closely linked to both immediate and recovery‐phase metabolic responses.^[^ ^39^ ^]^ Note that the correlation between the reference and estimated SBPs demonstrates a strong linearity, evidenced by an R‐value of 0.95 and MAD of 3.80 mmHg for all the sequential stages (Figure 5c; Figure S18, Supporting Information). The Bland–Altman plot also reveals a mean estimation error of 0.98 mmHg with 95% confidence intervals, indicating an excellent fit between the reference and estimated SBP values (Figure 5d).

The HS‐PPG further demonstrates detailed physiological parameters for continuous blood pressure monitoring during rest, exercise, and recovery (**Table** **1**; Table S1 and Figure S19, Supporting Information). The HS‐PPG sensor provides unique insights into SBP values including SBP variability, slope, and response during exercise, which are unattainable by a cuff‐based BP sensor. The SBP responses, SBP recovery rate, and recovery time also exhibit less deviation from the HS‐PPG sensor, providing more accurate and continuous BP monitoring. The recovery time from the HS‐PPG sensor shows an average of 4.20 min, while also providing precise measurements from the HR sensor and respiration calorimeter. As a result, the wearable HS‐PPG sensor allows continuous, sensitive BP monitoring with immediate and accurate responses during exercise and recovery.

**Table 1:** Physiological parameters during exercise and recovery. HS‐PPG acquires SBP variability, slope, and response values during exercise, which are unattainable with a cuff‐based BP sensor. In addition, the SBP recovery rate and recovery time for HS‐PPG exhibit less deviation and offer more precise values compared to those from the HR sensor and respiration calorimeter. Note that Δ_HSPPG‐cBP_ is the difference value between cuff‐based BP sensor and HS‐PPG module divided by the value of HS‐PPG.

Physiological parameter^a)^	AVG	SD	sensor	Δ_HSPPG‐cBP_
SBP variability during exercise	1.70 mmHg	0.73 mmHg	HS‐PPG	–
SBP slope during exercise	1.22	0.09	HS‐PPG	–
exercise‐induced SBP response	34.50 mmHg	8.17 mmHg	HS‐PPG	–
SBP variability during rest	2.03 mmHg	0.77 mmHg	HS‐PPG	
	2.64 mmHg	0.92 mmHg	Cuff BP	30.05%
SBP variability during recovery	1.98 mmHg	1.16 mmHg	HS‐PPG	
	2.60 mmHg	0.92 mmHg	Cuff BP	31.31%
SBP recovery rate	0.85	0.04	HS‐PPG	
	0.89	0.06	Cuff BP	4.71%
recovery time (SBP)	4.20 min	0.92 min	HS‐PPG	
	3.90 min	1.20 min	Cuff BP	−7.14%
recovery time (HR)	4.20 min	1.62 min	HR sensor	
recovery time (RER)	4.40 min	1.51 min	respiration calorimeter	

^a)^ References: SBP variability during rest/exercise/recovery,^[^ ^43^ ^]^ SBP slope during exercise, recovery time (SBP),^[^ ^44^ ^]^ exercise‐induced SBP response,^[^ ^45^ ^]^ SBP recovery rate, recovery time (SBP),^[^ ^40^ ^]^ recovery time (RER)^[^ ^39^, ^41^, ^42^ ^]^

## 3 Conclusion

In summary, we have successfully demonstrated wearable HS‐PPG for continuous BP monitoring of exercise‐induced hypertension. The HS‐PPG module employs DFSIG‐µSPEC with high spectral resolution, utilizing the solid immersion grating in a broad operational range of 550–800 nm. The DFSIG‐µSPEC exhibits an average spectral resolution of 3.4 nm and relative sensitivity of over 60% of the maximum. The HS‐PPG module was fully integrated with DFSIG‐µSPEC and a white LED in a wrist‐wearable configuration. The module measures HS‐PPG waveforms and precisely calculates aPTT values, and subsequently estimates BP level using the 2‐element Windkessel model. BP monitoring in normotensive subjects demonstrates strong correlations between BP values estimated by the HS‐PPG module and those obtained from a cuff‐based BP sensor, achieving high reliability with R‐values of 0.92 for DBP and 0.96 for SBP. In addition, continuous BP monitoring with exercise‐induced hypertension shows high estimation accuracy, with an R‐value of 0.95 confidence intervals of 95%. The continuous BP monitoring via HS‐PPG provides valuable cardiovascular insights, offering detailed physiological parameters related to SBP during exercise and recovery. This wearable optical platform can enhance resource efficiency, reduce healthcare costs, and advance precision medicine and proactive digital healthcare.

## 4 Experimental Section

### Monte–Carlo Ray Tracing

The optical model used for the HS‐PPG was based on the GPU‐accelerated 3D Monte–Carlo ray tracing.^[^ ^47^, ^48^ ^]^ The simulation domain was set to 16 mm × 16 mm × 5 mm to account for backscattering within the skin. For simplicity, a single‐layer human skin model was used with non‐dispersive scalar absorption coefficient of 1 cm^−1^, scattering coefficient of 25 cm^−1^, the Henyey–Greenstein anisotropy factor of 0.9, and relative refractive index of 1.4.^[^ ^47^, ^49^, ^50^ ^]^ The source‐detector separation was set to 3 mm, considering the LED size. The light source was modeled as a 0.1 mm square with a Lambertian angular distribution. The detector's NA was set to 0.2 to match the DFSIG‐µSPEC, and the diameter was set to 0.5 mm for simulation stability. The wavelength range was set from 550 to 800 nm with a flat spectral power to cover the entire operational range. A total of 109 photons were simulated, and the cross‐section of the source normalized photon flux was defined at 1.5 mm, i.e., the midpoint between the source and detector.

### BP Estimation

For SBP and DBP estimation, mean BP (MBP) and pulse pressure (PP) are first calculated with continuous aPTT value. Both MBP and PP are affected by the aPTT as follows;

(1) $$MBP=HR\left(\lambda \right)\cdot \left(k_{1}\cdot aPTT\left(\lambda \right)+b_{1}\right)$$

(2) $$PP=MBP\cdot \left(k_{2}\cdot \frac{t_{\tau }\left(\lambda \right)}{HR\left(\lambda \right)}+b_{2}\right)$$

where HR is heart rate, k_1_, k_2_, b_1_, b_2_ are the subject‐dependent variables in the simplified model. The time constant *t*_τ_(λ) at two‐element Windkessel model can be approximated as below;

(3) $$t_{\tau }\left(\lambda \right)\approx 0.63\cdot \left(P_{c}-DBP\right)$$

where *P_c_* is the pressure value at the closure of aortic valve. Here, *t*_τ_ is the constant for blood pressure estimation model which is related to the systemic vascular resistance (SVR) and arterial compliance (AC);

(4) $$t_{\tau }\left(\lambda \right)\approx SVR\left(\lambda \right)\cdot AC\left(\lambda \right)$$

And the relationship between MBP and PP can be expressed as the variables which related to the performance of the heart.^[^ ^46^ ^]^ MBP and PP can be written as

(5) MBP≈CO·SVR

(6) CO=SV·HR

where CO is the cardiac output and SV is the stroke volume where the blood volume per single beat. The AC as a change in volume by a change in blood pressure, can be expressed as

(7) $$PP=\frac{SV}{AC}$$

Combining equations above, the relationship between MBP, PP, and *t*_τ_ can be expressed as

(8) $$\frac{MBP}{PP}=HR\left(\lambda \right)\cdot t_{\tau }\left(\lambda \right)$$

To sum up, MBP, PP, k_1_, k_2_, b_1_, b_2_ can be expressed as two different formula. By inputting HR and aPTT values at 50 different wavelengths, MBP and PP are estimated from the slope and constant of the trend line at a single subject. SBP and DBP are then calculated by below equations;

(9) $$MBP=\frac{1}{3}\;SBP+\frac{2}{3}DBP$$

(10) PP=SBP−DBP

A simplified vascular model is used for monitoring of SBP and DBP levels through continuous aPTT values.

### Continuous BP Monitoring

Ten normotensive subjects without cardiovascular disease were recruited for BP estimation. The subjects were five men and five women aged between 20 and 25 years. The experimental setup consisted of three stages of rest, exercise, and recovery. All the subjects equipped four respective sensors; cuff‐based BP sensor (Hativ P30, Vuno) for reference BP, HS‐PPG module for estimated BP, HR sensor (Polar OH1, Polar Electro Oy), and calorimeter (K5, Cosmed). First, the subjects took a rest while sitting for 10 min. The BP sensor measured reference BP with 2 min‐intervals, and other sensors monitored estimated BP, HR, and RER with a 1 min‐interval. Next, the subjects started exercising at the ClimbMill (Shandong Minolta Fitness Equipment), and monitored BP, HR, and RER excluding the cuff‐based reference BP. The exercise step was performed at a speed of 72 stages min^−1^ for 10 min, and the exercise was stopped when HR reached 90% of the maximum or when subjects felt uncomfortable and voluntarily wanted to stop. Finally, the subjects come down from the ClimbMill, sit down and recover for 10 min, and monitored while wearing four respective sensors same as the resting step. All the experiment has been approved by the Korea Advanced Institute of Science and Technology Institutional Review Board (KAIST IRB, KH2023‐148), and all subjects gave informed consent before the experiment.

## Conflict of Interest

The authors declare no conflict of interest.

## Supporting information



Supporting Information

## Data Availability

The data that support the findings of this study are available from the corresponding author upon reasonable request.
