# Supplementary material for: Wearable Hyperspectral Photoplethysmography Allows Continuous Monitoring of Exercise‐Induced Hypertension
Source: Adv Sci (Weinh). 2025 Apr 25;12(22):2417625. doi: 10.1002/advs.202417625 (PMC12165084; doi:10.1002/advs.202417625)
Supplement: Supplementary file 1 — Supporting Information [file ADVS-12-2417625-s001.docx]

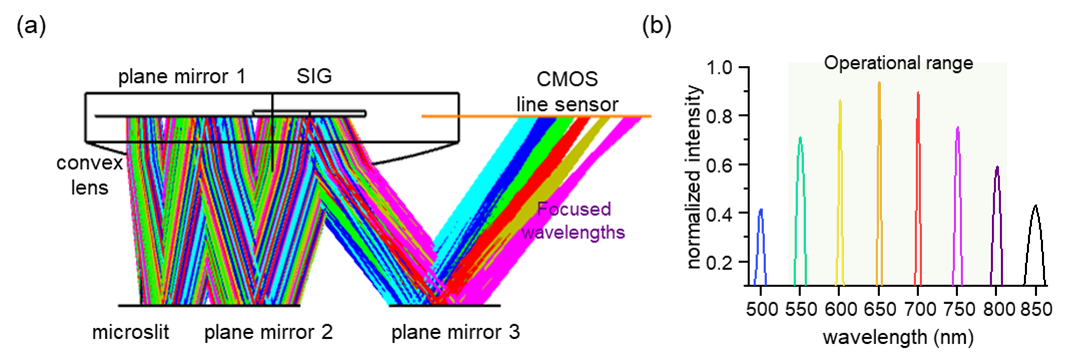


**Fig. S1 Optical design of DFSIG-μSPEC.** (a) Optical layout of the DFSIG-μSPEC using a ray-tracing software on Zemax OpticStudio. Light from 500 nm to 850 nm with 50 nm interval is focused on the CMOS line sensor. (b) Focal profiles on the image plane. The operational range of 550 nm to 800 nm is determined by the normalized spectral intensity above 60% of the maximum.


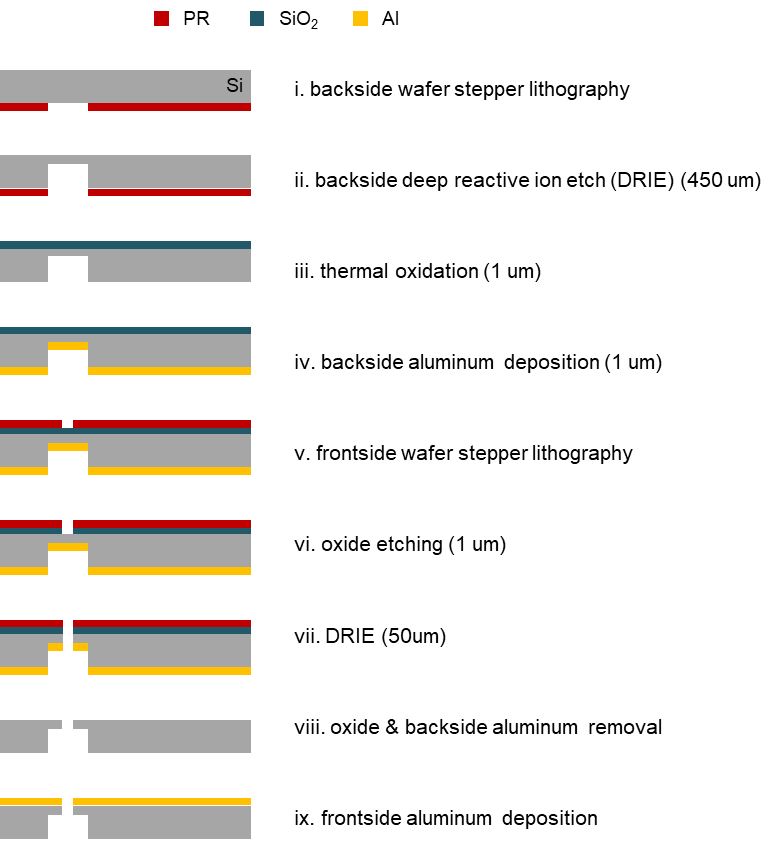


**Fig. S2 Microfabrication procedure of the silicon microslit.** A silicon microslit was fabricated by double-side DRIE, with a thickness of 450 μm (backside) and 50 μm (frontside), respectively.


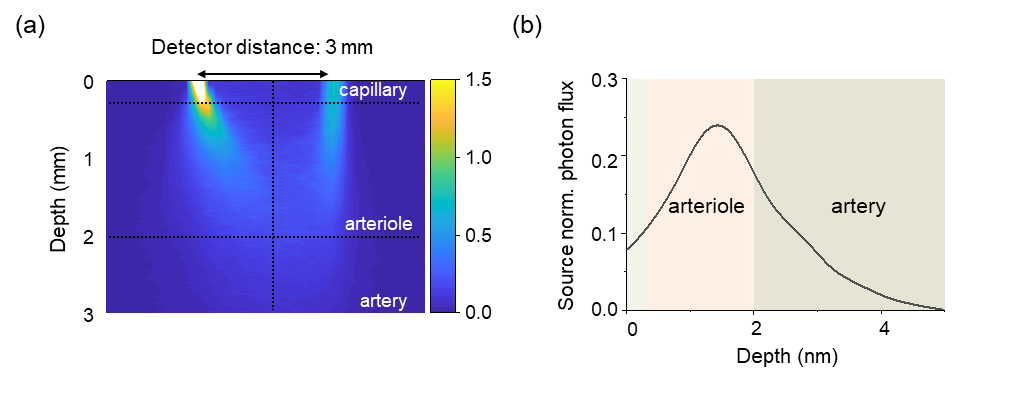


**Fig. S3 Monte-Carlo simulation results for HS-PPG waveform measurement.** (a) Simulation for evaluating detector distance. The distance between white LED and DFSIG-μSPEC was determined to be 3 mm, which has the maximum signal intensity at the arteriole layer. (b) Cross-section of source normalized photon flux at the midpoint between LED and DFSIG-μSPEC with different depth. With the detector distance at 3 mm, photon flux has the maximum value at the depth of 1.42 mm.


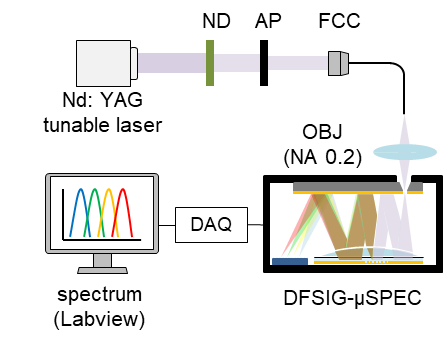


**Fig. S4 Experimental setup for measuring spectral resolution of DFSIG-μSPEC**. A hyperspectral light of 10 nm interval passes through ND filter, AP, FCC, OBJ, and then entered into DFSIG-μSPEC. The output spectra are visualized by using DAQ software.


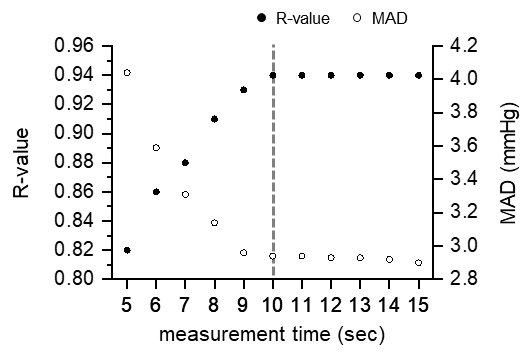


**Fig. S5. The estimation reliability for HS-PPG waveform measurement**. The R-value and MAD for different measurement time of HS-PPG waveform. The temporal resolution is determined to be 10 seconds, which has the most rapid time to reach maximum R-value and minimum MAD.


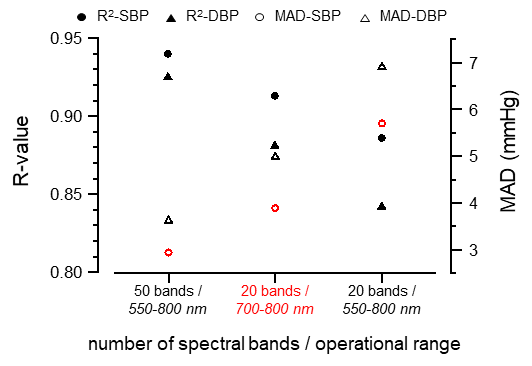


**Fig. S6. The estimation reliability for HS-PPG with different number of spectral bands and operational range.** HS-PPG with the operational range of over 700 nm has high R-values, with 0.91 of SBP and 0.83 of DBP. Also, the MADs are lower than IEEE standard level, with 3.89 *mmHg* of SBP and 4.98 *mmHg* of DBP. The HS-PPG module improves BP estimation reliability even with the negligible effect of the melanin absorption ^50^.


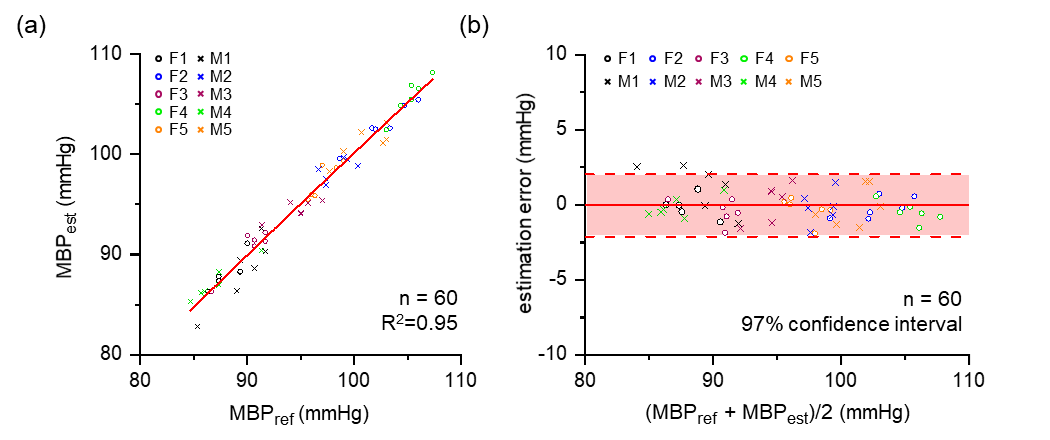


**Fig. S7. MBP in normotensive states.** (a) A strong linear correlation of MBP values from cuff-based BP sensor (MBP_ref_) and HS-PPG module (MBP_est_). MBPs show high linearity with an R-value of 0.95 and the MAD of 0.85 mmHg. (b) The Bland-Altman plot for MBP values. Two MBPs show a mean estimation error of -0.03 mmHg with the confidence intervals of 97%.


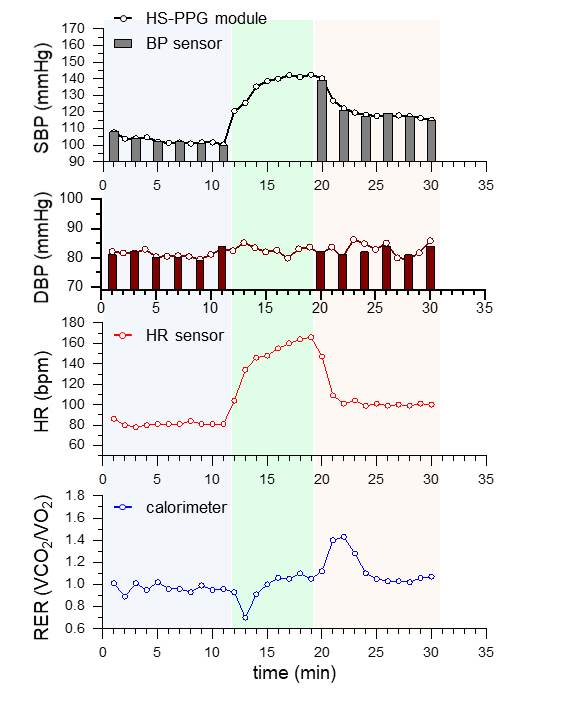


**Fig. S8 Raw data for clinical validation**. SBP, DBP, HR, and RER values from subject 1.


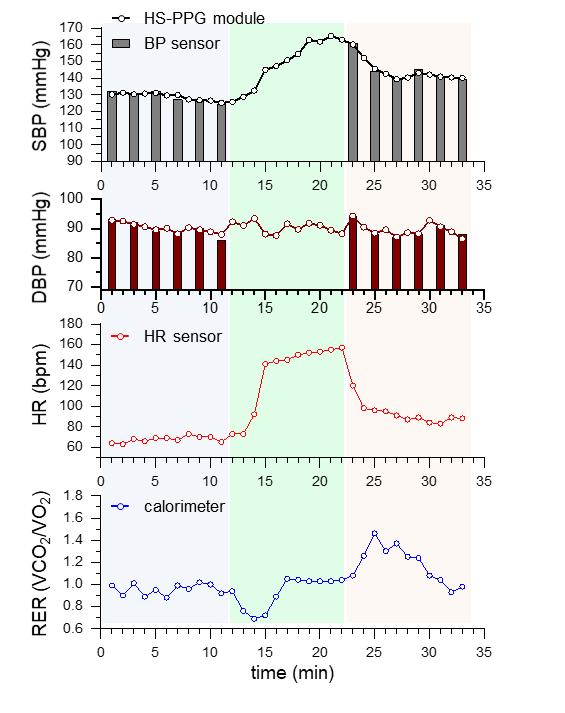


**Fig. S9 Raw data for clinical validation**. SBP, DBP, HR, and RER values from subject 2.


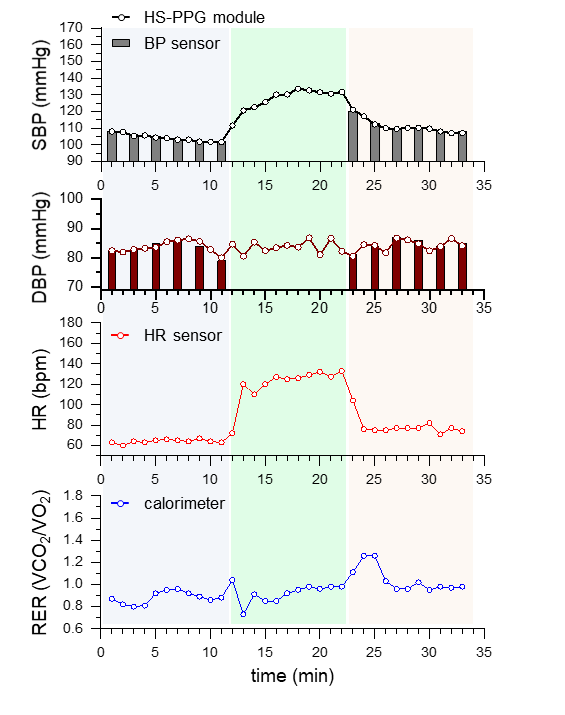


**Fig. S10 Raw data for clinical validation**. SBP, DBP, HR, and RER values from subject 3.


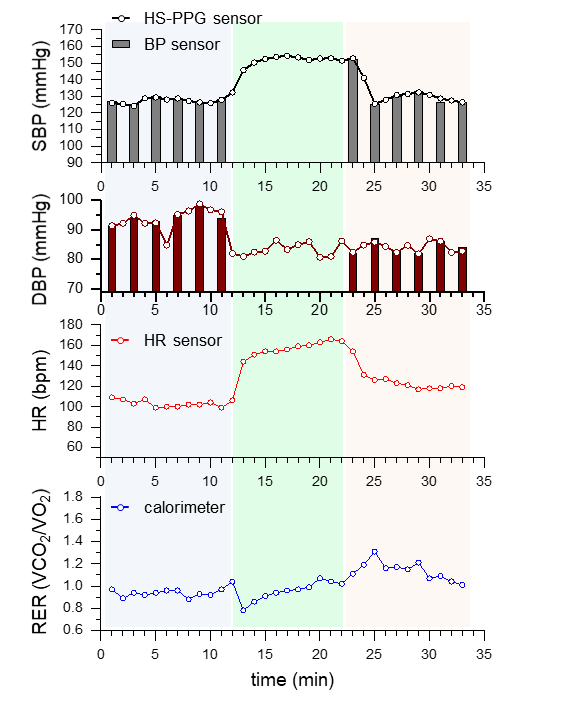


**Fig. S11 Raw data for clinical validation**. SBP, DBP, HR, and RER values from subject 4.


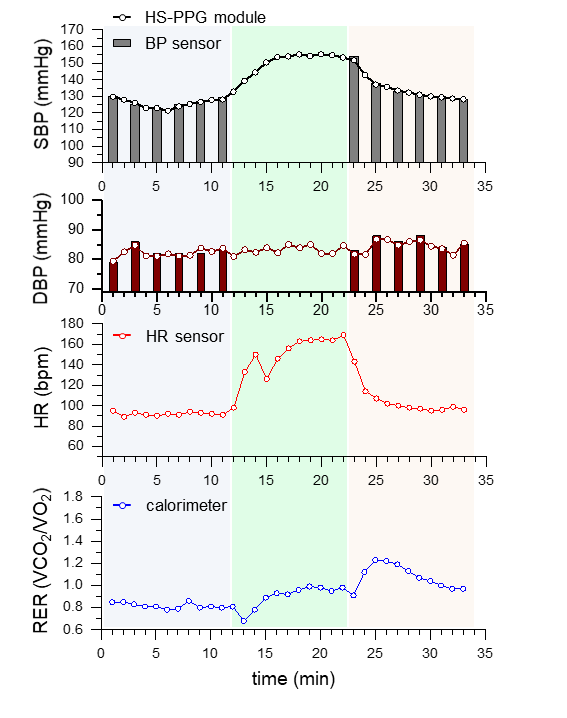


**Fig. S12 Raw data for clinical validation**. SBP, DBP, HR, and RER values from subject 5.


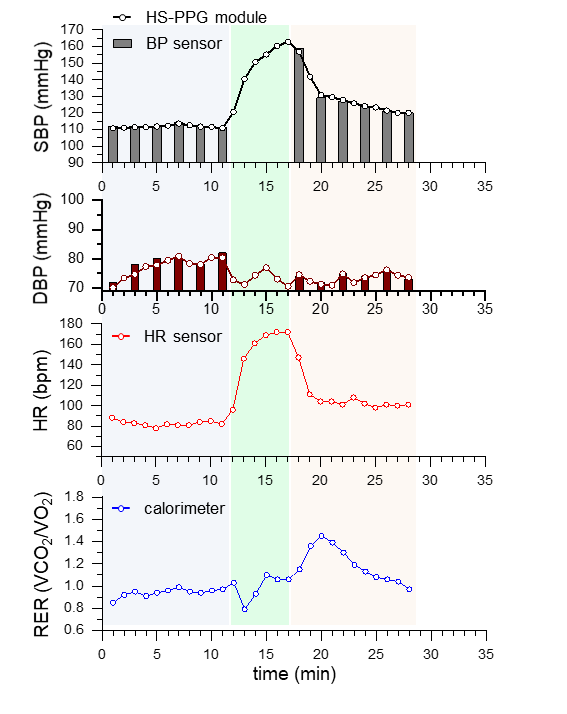


**Fig. S13 Raw data for clinical validation**. SBP, DBP, HR, and RER values from subject 6.


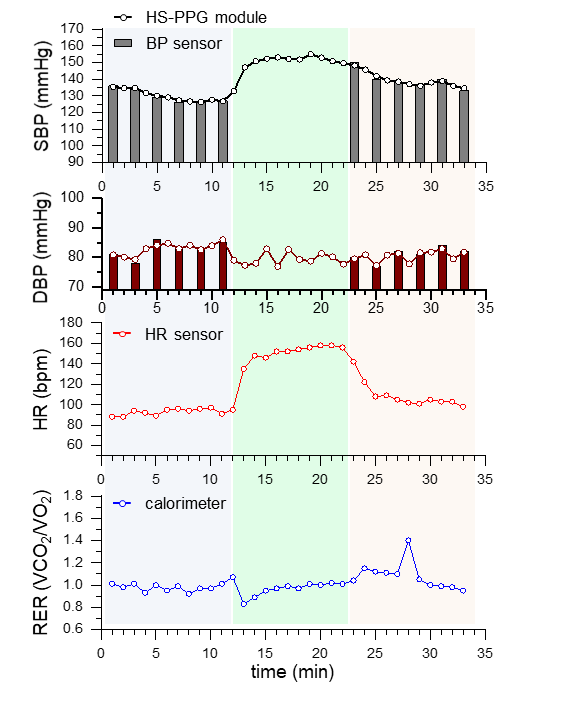


**Fig. S14 Raw data for clinical validation**. SBP, DBP, HR, and RER values from subject 7.


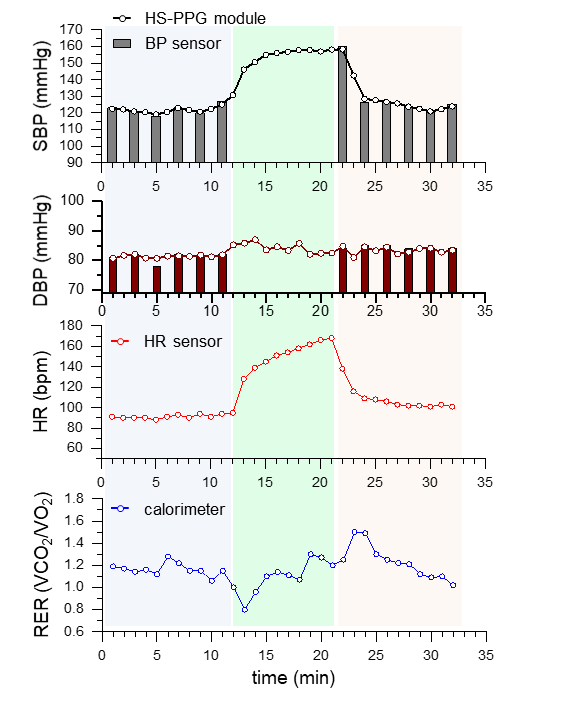


**Fig. S15 Raw data for clinical validation**. SBP, DBP, HR, and RER values from subject 8.


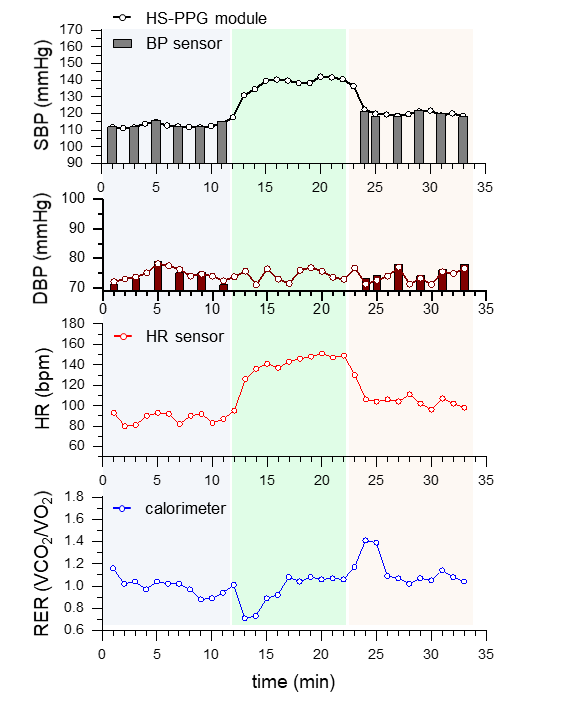


**Fig. S16 Raw data for clinical validation**. SBP, DBP, HR, and RER values from subject 9.


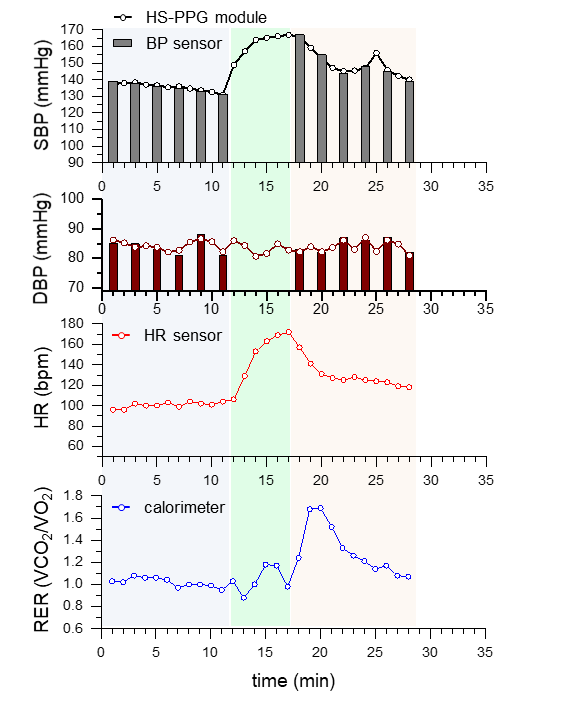


**Fig. S17 Raw data for clinical validation**. SBP, DBP, HR, and RER values from subject 10.


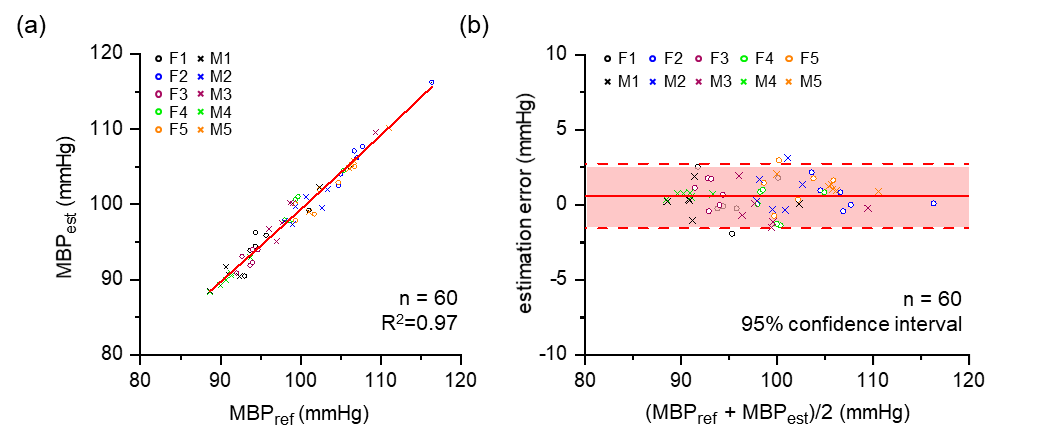


**Fig. S18. MBP in exercise-induced hypertension states.** (a) A strong linear correlation of MBP values from cuff-based BP sensor (MBP_ref_) and HS-PPG module (BMP_est_). MBPs show high linearity with an R-value of 0.97 and the MAD of 0.99 mmHg. (b) The Bland-Altman plot for MBP values. Two MBPs show a mean estimation error of 0.59 mmHg with the confidence intervals of 95%.

**Table S1. physiological parameters during rest, exercise and recovery**. The physiological parameters before, during, and after exercise were extracted from data measured by each sensor. The HS-PPG sensor acquires SBP variability, SBP slope, SBP response during exercise which is unattainable for cuff-based BP sensor. SBP recovery rate, recovery time, HR recovery rate, and RER slope show large deviation due to differences in the exercise capacity of each subject. Note that Δ_HSPPG-cBP_ is the difference value between cuff-based BP sensor and HS-PPG module divided by the value of HS-PPG.

| **physiological parameter** | **AVG** | **SD** | **sensor** | **Δ_HSPPG-cBP_** |
| --- | --- | --- | --- | --- |
| SBP variability during exercise | 1.70 mmHg | 0.73 mmHg | HS-PPG | - |
| SBP slope during exercise | 1.22 | 0.09 |  | - |
| exercise-induced SBP response | 34.50 mmHg | 8.17 mmHg |  | - |
| SBP response during recovery | 19.70 mmHg | 9.38 mmHg | Cuff BP | -20.37 % |
|  | 24.74 mmHg | 5.94 mmHg | HS-PPG |  |
| baseline SBP during rest | 120.17 mmHg | 11.12 mmHg | Cuff BP | -0.12 % |
|  | 120.02 mmHg | 11.09 mmHg | HS-PPG |  |
| SBP variability during rest | 2.64 mmHg | 0.92 mmHg | Cuff BP | -30.05 % |
|  | 2.03 mmHg | 0.77 mmHg | HS-PPG |  |
| SBP variability during recovery | 2.60 mmHg | 0.92 mmHg | Cuff BP | -31.31 % |
|  | 1.98 mmHg | 1.16 mmHg | HS-PPG |  |
| SBP recovery rate | 0.89 | 0.06 | Cuff BP | -4.71 % |
|  | 0.85 | 0.04 | HS-PPG |  |
| recovery time (SBP) | 3.90 min | 1.20 min | Cuff BP | 7.14 % |
|  | 4.20 min | 0.92 min | HS-PPG |  |
| HR recovery rate | 41.40 bpm | 16.05 bpm | HR sensor |  |
| recovery time (HR) | 4.20 min | 1.62 min |  |  |
| RER magnitude | 27.00 | 10.34 | respiration calorimeter |  |
| time to RER max | 102.0 sec | 27.50 sec |  |  |
| RER slope | 26.67 sec^-1^ | 7.75 sec^-1^ |  |  |
| recovery time (RER) | 4.40 min | 1.51 min |  |  |

* references: SBP variability during exercise/rest/recovery ^43^, SBP slope during exercise, recovery time ^44^, exercise-induced SBP response, SBP response during recovery ^45^, baseline SBP during rest ^42^, SBP recovery rate, recovery time, HR recovery rate, recovery time (HR) ^40^, RER magnitude, time to RER max, RER slope, recovery time (RER) ^39^


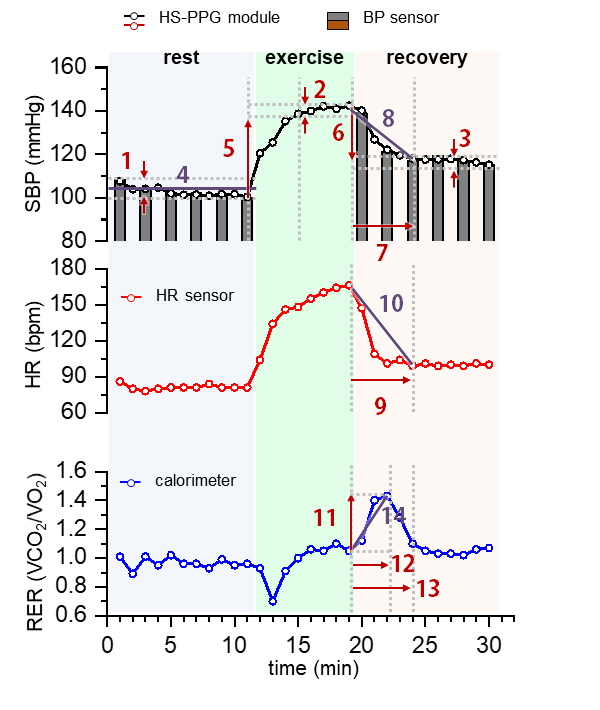


**Fig. S19. Physiological parameters acquired from SBP, HR and RER values.** Each physiological parameter is matched as follows: 1. SBP variability during rest / 2. SBP variability during exercise / 3. SBP variability during recovery / 4. SBP baseline during rest / 5. exercise-induced SBP response / 6. SBP response during recovery / 7. recovery time (SBP) / 8. SBP recovery rate / 9. recovery time (HR) / 10. HR recovery rate / 11. RER magnitude / 12. time to RER max / 13. recovery time (RER) / 14. RER slope.
